# Image acquisition as novel colonoscopic quality indicator: a single-center retrospective study

**DOI:** 10.3389/fonc.2023.1090464

**Published:** 2023-05-08

**Authors:** Ke Zhang, Abdiwahid Mohamed Bile, Xinyi Feng, Yemin Xu, Yaoyao Li, Qiang She, Guiqing Li, Jian Wu, Weiming Xiao, Yanbing Ding, Bin Deng

**Affiliations:** ^1^ Department of Gastroenterology, Affiliated Hospital of Yangzhou University, Yangzhou, China; ^2^ Graduate School, Dalian Medical University, Dalian, ;China; ^3^ Medical College, Yangzhou University, Yangzhou, China

**Keywords:** colonoscopy, quality, adenoma detection rate, polyp detection rate, photodocumentation

## Abstract

**Purpose:**

In order to reduce the incidence and mortality of colorectal cancer, improving the quality of colonoscopy is the top priority. At present, the adenoma detection rate is the most used index to evaluate the quality of colonoscopy. So, we further verified the relevant factors influencing the quality of colonoscopy and found out the novel quality indicators by studying the relationship between the influencing factors and the adenoma detection rate.

**Materials/methods:**

The study included 3824 cases of colonoscopy from January to December 2020. We retrospectively recorded the age and sex of the subjects; the number, size, and histological features of lesions; withdrawal time and the number of images acquired during colonoscopy. We analyzed the associated factors affecting adenoma and polyp detection, and verified their effectiveness with both univariate and multivariate logistic regression analyses.

**Results:**

Logistic regression analyses showed that gender, age, withdrawal time and the number of images acquired during colonoscopy could serve as independent predictors of adenoma/polyp detection rate. In addition, adenoma detection rate (25.36% vs. 14.29%) and polyp detection rate (53.99% vs. 34.42%) showed a marked increase when the number of images taken during colonoscopy was ≥29 (*P*<0.001).

**Conclusions:**

Gender, age, withdrawal time and the number of images acquired during colonoscopy are influencing factors for the detection of colorectal adenomas and polyps. And we can gain higher adenoma/polyp detection rate when endoscopists capture more colonoscopic images.

## Introduction

Colorectal cancer (CRC) has high incidence and accounts for roughly 10% of all cancer diagnoses and cancer-related deaths globally each year (1). Population-based screening is an important means of preventing CRC. The population-based screening and early detection program introduced in the United States in the 1990s had an impact on the incidence and mortality of CRC, which showed a decreasing trend (2).

Many CRC screening methods currently exist, but definitive diagnosis still depends on colonoscopy (3). Colonoscopy plays an increasingly important role in CRC prevention and has become a more common screening test for colorectal neoplasia (4, 5). It provides a direct visualization of the whole colon from the rectum to the cecum and even the anus and allows the histological evaluation of any abnormal endoscopic findings, as well as the complete removal of many precancerous lesion. According to the long-term follow-up of patients after colonoscopic polypectomy, early detection, early intervention, and long-term monitoring can remarkably reduce the incidence of CRC (6, 7). The wide application of colonoscopy has promoted the extensive research on the quality improvement of colonoscopy in recent years.

In fact, observational indicators are used to evaluate the quality of colonoscopy, especially in the early identification and intervention of tumors. These indicators including bowel preparation, cecal intubation rate (CIR), adenoma detection rate (ADR), polyp detection rate (PDR), rectal retroflection, withdrawal time, sedation practice and comfort level, annual procedure quantity. Among them, ADR is one of the most commonly used evaluation indicators. ADR, which is dependent on small adenomas, as they account for most of the adenomas detected during colonoscopy, has been the key point of most studies on CRC screening and has found remarkable differences between endoscopists (8–10). Improving ADR is believed to improve colonoscopy performance to reduce the morbidity and mortality of interval cancers (11, 12). Many methods have been developed to improve ADR (13). For example, Barclay found that a longer withdrawal time (>6 min) increases the detection rates of polyps and advanced tumors (14). Studies demonstrated that divided-dose bowel preparations increase ADR (15, 16). All of these parameters are artificially controllable factors during colonoscopy, but whether unknown factors may influence the ability of colonoscopy to detect lesions is unclear, such as pictures collection during colonoscopy. The images acquired during colonoscopy are the most intuitive evidence for the acquisition of colonoscopy results. Therefore, we hypothesized that the number of colonoscopy images acquired is also a factor that influences the quality of colonoscopy. We further verified the relevant factors influencing the quality of colonoscopy by studying the relationship between the influencing factors and the adenoma detection rate.

## Patients and methods

### Study population

**Figure 1:**
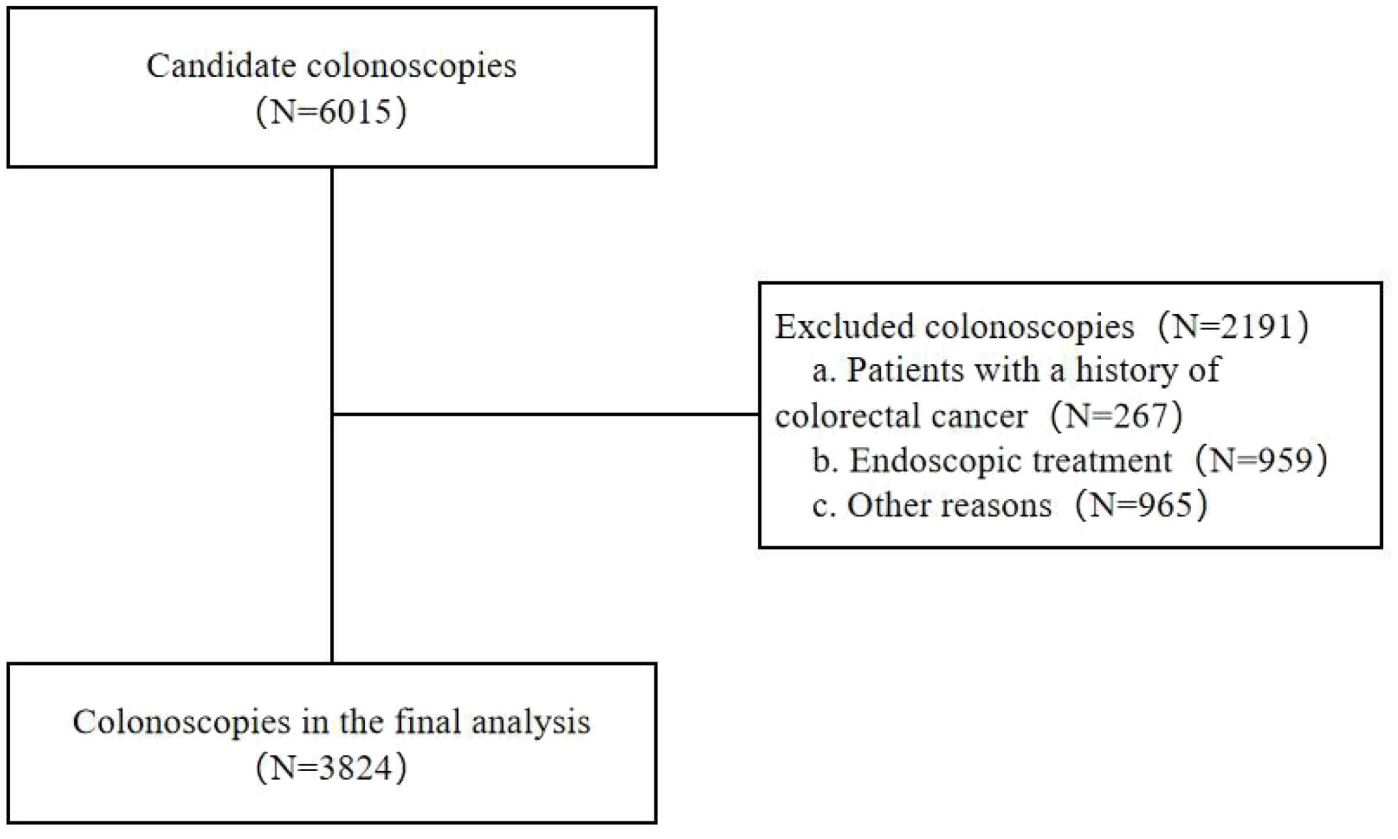
Study flow diagram and patients demographics.

Subjects who underwent colonoscopy at the Gastroscopy Center of the Affiliated Hospital of Yangzhou University from January to December 2020 were enrolled. The following inclusion criteria were applied: (1) subjects’ age ≥18 years; (2) subjects underwent colonoscopy for the first time. The exclusion criteria listed below were applied: (1) subjects with a personal history of CRC or colorectal resection; (2) pregnant or lactating women; (3) subjects with severe systemic diseases, mental disorders, and other diseases that might interfere with the assessment of the examination; (4) colonoscopies that were discontinued because of poor bowel preparation or other reasons; and (5) colonoscopies performed by endoscopists with a minimal number of operations per year (annual number of colonoscopies performed <500) or those with insufficient experience in colonoscopy (number of years of activity as endoscopist <3) (**Figure 1**). The Yangzhou University Affiliated Hospital’s Ethics Committee approved this study (No. 2021-YKL06-09-004). The need for informed consent was waived due to the retrospective nature of this study.

### Study procedures

Sixteen endoscopists in this study had dedicated, hands-on instruction for colonoscopy. All colonoscopies were performed in a hospital outpatient endoscopy center under venous anesthesia. We recorded the subjects’ age and sex, cecal intubation, withdrawal time and the number of images acquired during colonoscopy, as well as the number, size, location, and histological description of the lesions detected during colonoscopy. In the process of recording the number of colonoscopy pictures taken, when there are repeated pictures taken, i.e. the same pictures are taken two or more times, only one is recorded. When there were other errors, i.e. the pictures collected were blurred, in which case these pictures would be excluded from the study. The standard bowel preparation was a 3-liter oral lavage with polyethylene glycol electrolyte solution and dimeticone.

### Statistical analysis

Intergroup differences were compared using Student’s t-test. Chi-square test was used to analyze categorical data. Data were expressed as mean ± standard deviation. Multiple logistic regression analysis was used to determine the possible factors affecting lesion detection. Statistical Product and Service Solutions (SPSS) 22.0 software (SPSS Inc., Chicago, IL, USA) was used for statistical processing. Statistical significance was defined by *P ≤* 0.05. No guideline has been established for the number of images acquired during colonoscopy, thus, when we analyzed the number of images taken during colonoscopy, the median number of images acquired in all subjects was used, 29 as the basis for grouping.

## Results

### Study population

The baseline characteristics of subjects are illustrated in **Table 1**. Based on the inclusion and exclusion criteria, 3824 subjects were selected from 6015 subjects for inclusion in the study. The average age of the subjects included was 53.15 years, and 57.51% were male. Colorectal polyps were more frequently observed in participants who were older, male, with a longer withdrawal time and a higher number of images during colonoscopy (*P*<0.001).

**Table 1:** Clinical characteristics of the subjects.

Variables	Subjects without colorectal polyps (N=2090)	Subjects with colorectal polyps (N=1538)	*p* ^#^ value
Male Sex (no. [%])	1022 (48.90)	1049 (68.21)	<0.001**
Age (years)	50.45 ± 11.99	56.26 ± 11.53	<0.001**
Withdrawal Time(mins)	3.83 ± 1.99	8.69 ± 7.64	<0.001**
Cecal Intubation (no. [%])	2012 (96.27)	1439 (93.56)	<0.001**
^a^No.	28.31 ± 10.14	33.90 ± 13.85	<0.001**

^**^p<0.001; ^#^p value from χ^2^ test (or Fisher’s exact test, as appropriate) or t-test; a: number of images acquired during colonoscopy.

### Outcome measures

#### Relevant factors for lesion detection

The influence of various factors (age, sex, withdrawal time and the number of images taken during colonoscopy) on lesion detection rate was studied (**Table 2**). The subjects were divided into two groups depending on the presence or absence of colorectal adenomas and polyps. The ADR (23.40% vs. 9.08%) and PDR (52.93% vs. 33.35%) of subjects aged ≥45 years were significantly higher than those aged <45 years (*P*<0.001), and males were higher than females (*P*<0.001). The ADR (61.02% vs. 10.88%) and PDR (78.96% vs. 27.02%) are remarkably greater among endoscopists with a mean withdrawal time of ≥6 minutes and a higher number of images taken during colonoscopy (*P*<0.001).

**Table 2:** Correlation between different factors and the detection of adenomas and polyps.

	ADR(%)	*p* value	PDR(%)	*p* value
Sex		<0.001^**^		<0.001^**^
Male	23.92	–	52.93	–
Female	14.83	–	33.35	–
Age (years)		<0.001^**^		<0.001^**^
≥ 45	23.40	–	49.97	–
< 45	9.08	–	27.02	–
Withdrawal Time(mins)		<0.001^**^		<0.001^**^
≥ 6	61.02	–	78.96	–
< 6	10.88	–	27.02	–
^a^No.		<0.001^**^		<0.001^**^
≥ 29	25.36	–	53.99	–
< 29	14.29	–	34.42	–

^**^p<0.001. a: number of images acquired during colonoscopy. ADR = adenoma detection rate, PDR = polyp detection rate.

Finally, in the univariate analyses, the odds of detecting an adenoma in women were 54.6% of those in men. People aged ≥45 years were more than three times as likely to develop adenomas as those aged <45 years. As the number of images collected during colonoscopy increased, ADR increased approximately 2-fold. Multivariate analyses showed that gender, age, withdrawal time and the number of images acquired during colonoscopy could serve as independent predictors of ADR (**Table 3**). Similar results were obtained in the analysis of polyp detection (**Table 4**).

**Table 3:** Logistic regression analysis of relevant risk factors that may influence adenoma detection.

Risk Factors	Univariate Analyses		Multivariate Analyses	
	OR (95%CI)	*p* value	OR (95%CI)	*p* value
Gender	0.546 (0.457–0.651)	<0.001^**^	0.613 (0.508–0.740)	<0.001^**^
Age	3.229 (2.489–4.190)	<0.001^**^	2.810 (2.145–3.680)	<0.001^**^
Withdrawal Time	4.996 (4.193–5.952)	<0.001^**^	4.406 (3.374–4.853)	<0.001^**^
^a^No.	2.037 (1.714–2.421)	<0.001^**^	1.542 (1.281–1.855)	<0.001^**^

^**^p<0.001; a: number of images acquired during colonoscopy.

**Table 4:** Logistic regression analysis of the relevant risk factors that may influence polyp detection.

Risk Factors	Univariate Analyses		Multivariate Analyses	
	OR (95%CI)	*p* value	OR (95%CI)	*p* value
Gender	0.446 (0.389–0.512)	<0.001^**^	0.451 (0.385–0.529)	<0.001^**^
Age	2.630 (2.219–3.118)	<0.001^**^	2.519 (2.074–3.060)	<0.001^**^
Withdrawal Time	10.136 (8.545–12.024)	<0.001^**^	8.712 (7.297–10.400)	<0.001^**^
^a^No.	2.114 (1.849–2.418)	<0.001^**^	1.575 (1.347–1.841)	<0.001^**^

^*^p<0.05, ^**^p<0.001; ^a^number of images acquired during colonoscopy.

#### Effect of the photodocumentation of colonoscopy on lesion detection

Based on the above results, we further specifically analyzed the impact of picture recording on the quality of colonoscopy. **Table 5** describes the effect of the number of images acquired during colonoscopy on the detection of size, number, and pathology of lesions. According to the number of images, subjects were divided into two groups (1991 [≥29] vs. 1833 [<29]), and the difference in ADR (25.36% vs. 14.29%) and PDR (53.99% vs. 34.42%) between the two groups was significant (*P*<0.001). Excluding normal subjects, subjects in the lesion group were divided into two groups according to the number of images acquired during colonoscopy (1075 [≥29] vs. 631 [<29]). The difference in the detection rate of polyps with ≥6 mm diameter was significant between the two groups (*P*<0.05), and the difference in the detection rate of ≥3 polyps between the two groups was significant (*P*<0.001). The difference in the detection rates between nonneoplastic polyps and neoplastic polyps was also significant (*P<*0.05).

**Table 5:** Effect of the number of images acquired during colonoscopy on lesion detection.

	^a^No. ≥ 29	^a^No. < 29	*p* value
ADR (%)	505 (25.36%)	262 (14.29%)	<0.001^**^
PDR (%)	1075 (53.99%)	631 (34.42%)	<0.001^**^
Polyp size			0.011^*^
<6 mm	583	382	–
≥6 mm	492	249	–
Number of Polyps			<0.001^**^
≤2	633	446	–
>2	442	185	–
Histological Features of Polyps			0.029^*^
Nonneoplastic Polyps	570	369	–
Neoplastic Polyps	505	262	–

^*^p<0.05, ^**^p<0.001; a: number of images acquired during colonoscopy. ADR = adenoma detection rate, PDR = polyp detection rate.

#### Detection of lesions in individual colonic segments

A strong correlation was found between the number of images acquired at each colorectal site and the detection rate of lesions (**Table 6**). Similarly, the median number of images acquired at different sites in the colon across all subjects was used as the cutoff. In the cecum and rectum, ADR and PDR were remarkably higher when the number of images acquired in each colonic segment was ≥3 compared with <3. In the ascending colon, transverse colon, descending colon, and sigmoid colon, ADR and PDR were remarkably higher when the number of images acquired in each colonic segment was ≥4. However, with the exception of the ascending and descending colons, no substantial differences were found between the two groups in the detection of large polyps (≥6 mm diameter). Moreover, no considerable difference was found between the two groups in terms of polyp number or polyp histopathology.

**Table 6:** Effect of the number of images acquired in individual colonic segments during colonoscopy on lesion detection.

		ADR (%)	*p* value	PDR (%)	*p* value	Size of Polyps		*p* value	Number of Polyps		*p* value	Histological Features of Polyps		*p* value
						≥6 mm	<6 mm		≤2	>2		Nonneoplastic Polyps	Neoplastic Polyps	
Cecum			<0.001^**^		<0.001^**^			0.235			0.826			0.857
	^a^No. ≥ 3	2.22	–	4.81	–	36	68	–	82	22	–	56	48	–
	^a^No. < 3	0.42	–	0.96	–	8	8	–	13	3	–	9	7	–
Ascending Colon			<0.001^**^		<0.001^**^			0.001^*^			0.079			0.721
	^a^No. ≥ 4	5.81	–	11.86	–	146	95	–	197	44	–	123	118	–
	^a^No. < 4	1.90	–	4.07	–	28	45	–	66	7	–	39	34	–
Transverse Colon			<0.001^**^		<0.001^**^			0.234			0.419			0.670
	^a^No. ≥ 4	6.28	–	13.81	–	162	147	–	264	45	–	164	145	–
	^a^No. < 4	1.65	–	3.76	–	25	32	–	51	6	–	32	25	–
Descending Colon			<0.001^**^		<0.001^**^			0.016^*^			0.235			0.406
	^a^No. ≥ 4	5.22	–	12.72	–	146	132	–	237	41	–	146	114	–
	^a^No. < 4	1.22	–	3.48	–	20	37	–	45	12	–	37	20	–
Sigmoid Colon			<0.001^**^		<0.001^**^			0.978			0.595			0.235
	^a^No. ≥ 4	7.89	–	21.78	–	212	315	–	376	151	–	336	191	–
	^a^No. < 4	2.35	–	7.76	–	44	65	–	75	34	–	76	33	–
Rectum			<0.001^**^		<0.001^**^			0.583			0.977			0.302
	^a^No. ≥3	3.43	–	18.92	–	117	385	–	299	203	–	411	91	–
	^a^No.<	1.20	–	8.63	–	21	80	–	60	41	–	87	14	–

^**^p<0.001; a: number of images acquired during colonoscopy. ADR = adenoma detection rate, PDR = polyp detection rate.

## Discussion

Colonoscopy is the most common tool in CRC screening. It provides the chance to detect and remove benign lesions before the conditions deteriorate (14). ADR is the most commonly used marker for measuring colonoscopy quality and is used as an observation indicator to evaluate whether a new technology or technique improves the quality of colonoscopy (17). Based on our study, gender, age, withdrawal time and the number of images acquired during colonoscopy could serve as independent predictors of ADR.

In the past few decades, CRC cases have increased dramatically in the United States and other high-income countries. The incidence rate of CRC is 30% higher in men than in women, which may be related to male androgen levels (18, 19). According to our study, compared with women, men have a higher ADR, which is also consistent with previous studies. Therefore, we believe that males should pay more attention to colorectal cancer screening activities.

The 2021 American College of Gastroenterology screening guidelines also recommend CRC screening in average-risk population among ages 45–49 to decrease the incidence of advanced adenomas and carcinoma (3). Previously, in 2018, the American Cancer Society also published guidelines with a recommendation to reduce the initiation age for CRC screening in average-risk individuals from 50 years to 45 years and that starting screening at age 45 would result in a gain of approximately 25 additional life years per 1,000 individuals screened as compared with age 50 (20). Based on our findings, ADR and PDR substantially increased in subjects older than 45 years. Therefore, broadening the CRC screening population would be suitable.

According to a study involving 12 endoscopists, their analysis of screening colonoscopy in average-risk individuals found remarkable differences in the detection rates of lesions among endoscopists. Their results also showed that adequate withdrawal time can considerably improve colonoscopy quality (8). Shaukat et al. concluded that the incidence of interval cancer can be reduced by appropriately prolonging the withdrawal time during colonoscopy (21). Similarly, the increased withdrawal time also improved the ADR in our study. However, in normal subjects, their average withdrawal time was low and did not reach the guideline recommended time (3, 22), which requires further improvement later on.

In addition, we report for the first time in this study the effect of the number of images acquired taken during colonoscopy on colonoscopy quality in outpatients. Similar to withdrawal time, increasing the number of images acquired during colonoscopy suggests a more careful examination of the mucosa during colonoscopy and increases the chance of detecting lesions. The photodocumentation of cecal intubation had nominal effects on ADR and PDR. Acquiring more endoscopic images were more likely to demonstrate cecal intubation. Although their results did not reach statistical significance, the ADR and PDR of photographically confirmed colonoscopies were higher than those of deficiently photodocumented cases (23). Our results suggest that a difference in the number of images acquired during colonoscopy contributes to differences in the detection rates of lesions. In our study, ADR (25.36% vs. 14.29%) was significantly and markedly increased when the number of images taken during colonoscopy was ≥29. The resulting ADR was low, and the true ADR would be higher than our final ADR, because a large number of patients undergoing endoscopic treatment were initially excluded. In fact, the photodocumentation of abnormalities detected during colonoscopy has become universal. The habits of individual endoscopic operators in taking photos during colonoscopy vary, and the conception of images taken at normal sites, some prominent sites, and where abnormal lesions were present varies and may depend on the psychological state of the operator, which results in large differences in the number of drawings left. Our results suggest that the increased number of images acquired during colonoscopy increases the likelihood of detecting lesions and thus improves the quality of colonoscopy. However, whether this factor reduces the incidence and mortality of CRC is unclear, and future studies on photodocumentation during colonoscopy are warranted.

In our study, PDR paralleled ADR in trend, and the differences were significant. Most CRCs develop within adenomatous or serrated polyps, and the disruption of the polyp-to-cancer sequence prevents CRC progression. The increased detection and removal of colorectal polyps by colonoscopy is associated with a reduction in the incidence of advanced adenomas, carcinoma, and mortality from CRC (24). Briefly, our study results support the idea that the number of images acquired during colonoscopy correlates with the detection of polyps, and the results provide an opportunity for polypotomy, which may then reduce the incidence and mortality of CRC.

The ultimate aim of colonoscopy screening is to prevent CRC. Advanced adenomas in particular are more prone to develop into malignant diseases (25). According to the definition of the US Multi-Society Task Force on Colorectal Cancer, an advanced neoplasm is defined as an adenoma with a size of ≥10 mm, villous histology, or high-grade dysplasia. On follow-up after colonoscopy, patients found to have advanced adenomas are at increased risk of advanced neoplasia (26). However, the incidence of carcinoma is higher for lesions ≥6 mm than for lesions ≤5 mm (27). And it is difficult to differentiate benign and advanced adenomas by colonoscopy only (27–29). Therefore, the most recent clinical practice guidelines for the management of colorectal polyps strongly recommend endoscopic resection for lesions ≥6 mm in size (30). Our results also showed that acquiring a greater number of images during colonoscopy is correlated with a higher detection rate of large lesions. This result has remarkable implications for CRC screening by colonoscopy.

Finally, our study has several limitations. On the one hand, the analysis was not adjusted for patient factors, such as sedation, family history of CRC, and smoking, which may have influenced the results. On the other hand, this study is a single-center study. Further multicenter studies are needed to further verify the impact of colonoscopy photodocumentation on colonoscopy quality.

In our study, it is the first to explore the effect of colonoscopy photodocumentation on ADR and PDR. Besides ADR, cecal intubation rate and withdrawal time, we think that the image recording of colonoscopy is a novel quality indicator of colonoscopy that has been neglected for a long time, which is worth considering in the future recommendations and guidelines for colonoscopy quality indicators and screening. We call on gastroenterologists to take more pictures during colonoscopy. Overall, no studies to date have demonstrated appropriate specifications for image capture during colonoscopies. We obtained a higher ADRs and PDRs when endoscopists acquired more colonoscopic images. But the effect of a different number of images acquired during colonoscopy on CRC prevention is unknown. Our study was a rudimentary investigation; therefore, benefit, universality and meanings for clinical practice must be determined by farther studies.

## Data availability statement

The raw data supporting the conclusions of this article will be made available by the authors, without undue reservation.

## Ethics statement

This article does not contain any studies with human or animal subjects. All procedures were carried out in compliance with the Helsinki Declaration (as revised in 2013). This study was approved by the Ethics Committee of the Affiliated Hospital of Yangzhou University (No. 2021-YKL06-09-004). The need for informed consent was waived due to the retrospective nature of this study.

## Author contributions

All authors listed have made a substantial, direct, and intellectual contribution to the work. KZ, AB, and XF contributed to the study design. KZ, AB, YX, and GL contributed to data collection. JW, WX, YD, and BD were responsible for checking the data. YL and QS were responsible for revising critically of the article for important intellectual content. KZ, AB, and BD contributed to statistical analysis and preparation of the manuscript. All authors contributed to manuscript revision, read, and approved the submitted version.
